# The complete chloroplast genome sequence of *Eriobotrya fragrans*

**DOI:** 10.1080/23802359.2019.1676675

**Published:** 2019-10-12

**Authors:** Zhanghong Dong, Shaohong Qu, Cheng Liu, Peng Ye, Peiyao Xin

**Affiliations:** a Key Laboratory of Forest Resources Conservation and Utilization in the Southwest Mountains of China Ministry of Education, Southwest Forestry University, Kunming, China;; b South and Southeast Asia Joint R&D Center of Economic Forest Full Industry Chain, Southwest Forestry University, Kunming, China;; c International Technological Cooperation Base of High Effective Economic Forestry Cultirating, Southwest Forestry University, Kunming, China

**Keywords:** *Eriobotrya*, genome, phylogenetic relationship

## Abstract

*Eriobotrya fragrans* Champion ex Bentham is a potential medicinal plant of the genus *Eriobotrya* Lindl in the family Rosaceae. To better determine its phylogenetic location with respect to the other Rosaceae species, the complete chloroplast genome of *E. fragrans* was sequenced. The whole *E. fragrans* chloroplast genome is 159,286 bp in length, consisting of a pair of inverted repeat (IR) regions of 26,343 bp, one large single-copy (LSC) region of 87,301 bp, and one small single-copy (SSC) region of 19,299 bp. The overall GC content of the whole chloroplast genome is 36.7%. Further, phylogenetic analysis using maximum likelihood with TVM + F+R2 model strongly supports the relationship: sisterhood of *E. fragrans* and *E. japonica*, followed by three species of *Pyrus* L.

*Eriobotrya fragrans* Champ ex Bentham, an evergreen shrub tree species, is widely distributed in Guangdong, Guangxi, and Tibet of South China (http://foc.iplant.cn/). Among *Eriobotrya* Lindl plants, *E. fragrans* owns the stronger photosynthesis ability than the other species (Lin 2007). *Eriobotrya fragrans* has been reported to contain secondary metabolite polyphenols and flavonoids with antimicroorganism and antioxdant activities (Hong et al. 2008, 2009; Lin et al. 2011). For a better understanding of the relationships of *E. fragrans* and other Rosaceae species, it is necessary to perform high-throughput sequencing approaches.

About four gram fresh young and healthy leaves of *E. fragrans* in Mengla County (Yunnan, China; Long. 101.2546 E, Lat. 21.9263N, 564 m) were picked for DNA extraction from modified CTAB method (Liu et al. 2005). The voucher was deposited at the Key Laboratory of Forest Resources Conservation and Utilization in the Southwest Mountains of China Ministry of Education, Southwest Forestry University (Accession no. SWFU-SY35360). The whole chloroplast genome was sequenced following Yang et al. (2014), and the long-range PCR was used for next-generation sequencing with nine pairs of universal primers. Taking the *E. japonica* as a reference (Huang 2019), the complete chloroplast genome of *E. fragran*s was assembled using GetOrganelle software (Jin et al. 2018), and the chloroplast genome was annotated in Geneious 8.1.3 (Biomatters Ltd, Auckland, New Zealand).

The *E. fragrans* chloroplast genome, with a length of 159,286 bp, was 149 bp larger than that of *E. japonica* (159,137 bp, KT633951). It was also 782 bp and 871 bp smaller than that of *Malus domestica* (160,068 bp, KY818915) and *Pyrus ussuriensis* (160,157 bp, MK172841). The chloroplast genome has the usual quadripartite structure, featuring a LSC region (large single-copy region 87,301 bp), a SSC region (small single-copy region 19,299 bp), and a pair of IR (inverted repeats 26,343 bp). The overall GC content is 36.7% (LSC, 34.5%; SSC, 30.3%; IR, 42.7%). The *E. fragrans* chloroplast genome encodes 129 genes, including 84 protein-coding genes, 37 tRNA genes, and 8 rRNA genes.

To determine the phylogenetic location of *E. fragrans* with respect to the other Rosaceae with fully sequenced chloroplast genomes, the complete *E. fragrans* chloroplast was used to reconstruct the phylogenetic relationships. With the chloroplast of *Pentactina rupicola* (Maleae, Rosaceae, JQ041763) as an out-group, 13 chloroplast genome sequences of Rosaceae, including *Chaenomeles japonica* (KT932966), *Cydonia oblonga* (KX499857), *Docynia delavayi* (KX499860), *E. fragrans*, *E. japonica*, *Mulas baccatawere* (KX499859), *M. domestica*, *M. prattii* (MH929090), *Pyrus hopeiensis* (MF521826), *P. ussuriensis*, *P. x bretschneideri* (KX450881), and *Sorbus torminalis* (KY457242), aligned by the MAFFT version 7 programme (Katoh and Standley 2013). A maximum-likelihood analysis based on the TVM + F+R2 model was performed with iqtree version 1.6.7 using 1000 bootstrap replicates (Nguyen et al. 2015). The phylogenetic tree reveals that *E. fragrans* and *E. japonica* is most closely related to *Pyrus ussuriensis*, *P. hopeiensis*, and *P. x bretschneideri* (Figure 1), which is in agreement with previous reports on the close relationships between the two genera (Xiang et al. 2019).

**Figure 1.**
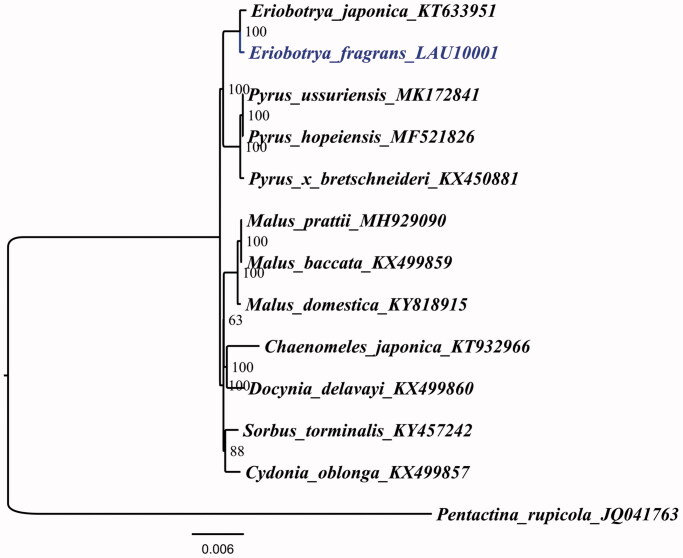
The ML phylogenetic tree for *E. fragrans* based on other 12 species (one in *Eriobotrya*, three in *Pyrus*, three in *Malus*, one in *Chaenomeles*, one in *Docynia*, one in *Sorbus*, one in *Cydonia*, and one in *Pentactina*) chloroplast genomes.

## Data Availability

The chloroplast data of the *E. fragrans* will be submitted to Rosaceae Chloroplast Genome Database (https://lcgdb.wordpress.com). Accession numbers are LAU10001.
